# Phase-coding memories in mind

**DOI:** 10.1371/journal.pbio.3000012

**Published:** 2018-08-29

**Authors:** Nicole Hakim, Edward K. Vogel

**Affiliations:** 1 Department of Psychology, University of Chicago, Chicago, Illinois, United States of America; 2 Institute for Mind and Biology, University of Chicago, Chicago, Illinois, United States of America; 3 Grossman Institute for Neuroscience, Quantitative Biology, and Human Behavior, University of Chicago, Chicago, Illinois, United States of America

## Abstract

Temporarily holding information in mind is an important part of many cognitive processes, such as reasoning and language. The amount of information that can be actively held “in mind” at any time is greatly limited—research suggests that we can only actively hold three or four pieces of information at once. A central question in cognitive neuroscience is how a system comprised of billions of neurons can actively maintain such a limited amount of information. A new study published in this issue of *PLOS Biology* by Bahramisharif and colleagues provides significant insights into this question.

## Introduction

**Fig 1:**
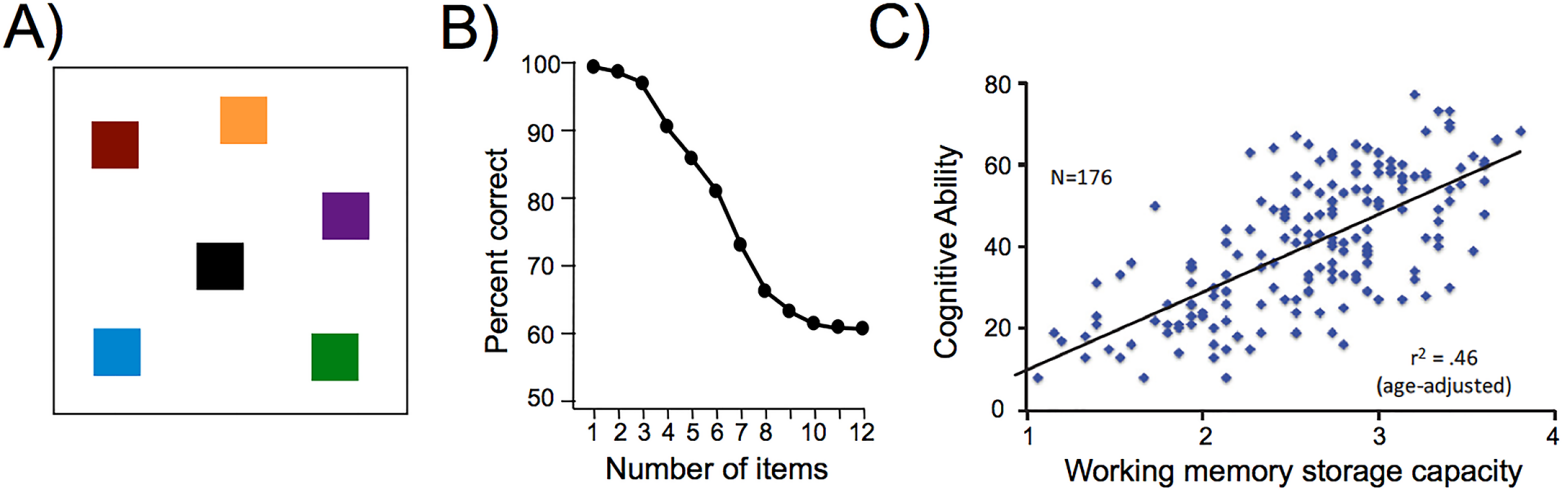
Working memory capacity and its relationship to cognitive abilities. Working memory capacity is often measured using a color change detection task (A). In this type of task, participants are asked to remember as many colored squares as possible over a brief delay. At test, one colored square reappears, and they have to determine whether the original square in that location was the same color as the newly presented square. People are very good at remembering up to three colored squares (B). However, performance begins to rapidly decline thereafter [4]. These results have been taken to suggest that typical working memory capacity is only about three to four objects. Additionally, there is substantial variability in the amount of information that individuals can hold in working memory. This variability in storage capacity is significantly correlated with cognitive abilities (C), as measured by the T score from the MATRICS Consensus Cognitive Battery [1].

The human brain is a powerful cognitive tool that has given rise to exceptional accomplishments: the development of life-saving vaccines, the establishment of civilizations, and the exploration of the moon. Our brains can store what seems to be a nearly infinite amount of information that we have learned over a lifetime, and we use this acquired knowledge to construct a coherent sense of the world around us. Despite these exceptional capabilities, the human cognitive system is greatly restricted in terms of how much information can be actively held “in mind” at a given moment. That is, despite the general feeling that we have awareness of much of our immediate environment, we have access to only a tiny sliver of it—sampling the physical world around us in bite-sized amounts of information from only three or four objects at a time (Fig 1). This “working memory” system allows us to temporarily hold information in a readily accessible state so that we can utilize it to perform complex cognitive tasks, such as reasoning and language. It is often considered to be the “mental workspace” for thinking. It is also a primary source of variation across humans in overall cognitive ability. An individual’s working memory capacity is a stable trait that is a strong predictor of his or her fluid intelligence, language skills, and scholastic achievement [1,2]. One central question in cognitive neuroscience is how a system comprised of billions of neurons with trillions of connections can manage to maintain only three or four pieces of information at any point in time. In this issue of *PLOS Biology*, a new study by Bahramisharif and colleagues [3] takes us one significant step closer to understanding how information in working memory is coded in brain activity and potentially why it is so limited.

## A forest of heterogeneous neurons

Revealing the mechanics of working memory at the neural level has been a vexing challenge for cognitive neuroscience. On the one hand, there is abundant evidence showing persistent neural activity related to performance on working memory tasks [5,6]. Many neurons in prefrontal and posterior parietal regions show sustained firing during the retention period for stimuli that match the selectivity of the cell, often referred to as delay activity. On the other hand, however, is decades of research showing that the responses of individual neurons that contribute to working memory performance are highly heterogeneous in terms of both selectivity and time course. Some neurons show sustained firing, while others show bursts of activity at seemingly random moments during the retention period [7]. This heterogeneity of inputs to the system greatly complicates any attempts to establish a coherent memory coding scheme [7–9]. However, the examination of single-neuron activity may provide too narrow of a view to determine how a large-scale brain-wide system, like working memory, operates. Instead, significant progress has been made when considering activity that is pooled across many heterogeneous individual neurons, giving rise to a “population-level” response. These population codes are well suited to accommodate massively heterogeneous inputs and yet still provide a stable and persistent signal that is suitable to guide working memory maintenance [10,11].

## Phase code model of working memory

When large groups of neurons fire together, they can create rhythmic electrical patterns. These neural oscillations can occur in multiple frequencies bands, including gamma (25–100 Hz), theta (4–8 Hz), and alpha (8–12 Hz) rhythms. This oscillatory activity is thought to facilitate communication between different areas of the brain [12] and has been proposed to play a central role in the maintenance of working memory representations. For example, previous research has suggested that gamma oscillations integrate individual features of memoranda held in working memory [13,14]. From this view, gamma helps to link together the various features of an object by integrating activity from several populations of neurons, each representing a given feature (e.g., color, orientation, position, etc.) by having them rhythmically fire together at that frequency. Additionally, theta and alpha oscillations have been shown to track allocation of attention [15] and to underlie attentional and executive coordination of sensory information [16–19]. Oscillations at these frequencies have additionally been shown to modulate neuronal excitability [20] by controlling neuronal firing, which could influence information held in working memory. While this neural synchronization proposal may provide an elegant solution to how the features of an object are bound together through coordinated firing patterns, it raises an even more important question: how can we hold multiple objects in memory at the same time? That is, how can multiple objects be represented in the same frequency band without accidentally confusing which feature goes with which object? One potential solution has been proposed by the Lisman/Idiart/Jensen (LIJ) model of cortical phase coding [21].

**Fig 2:**
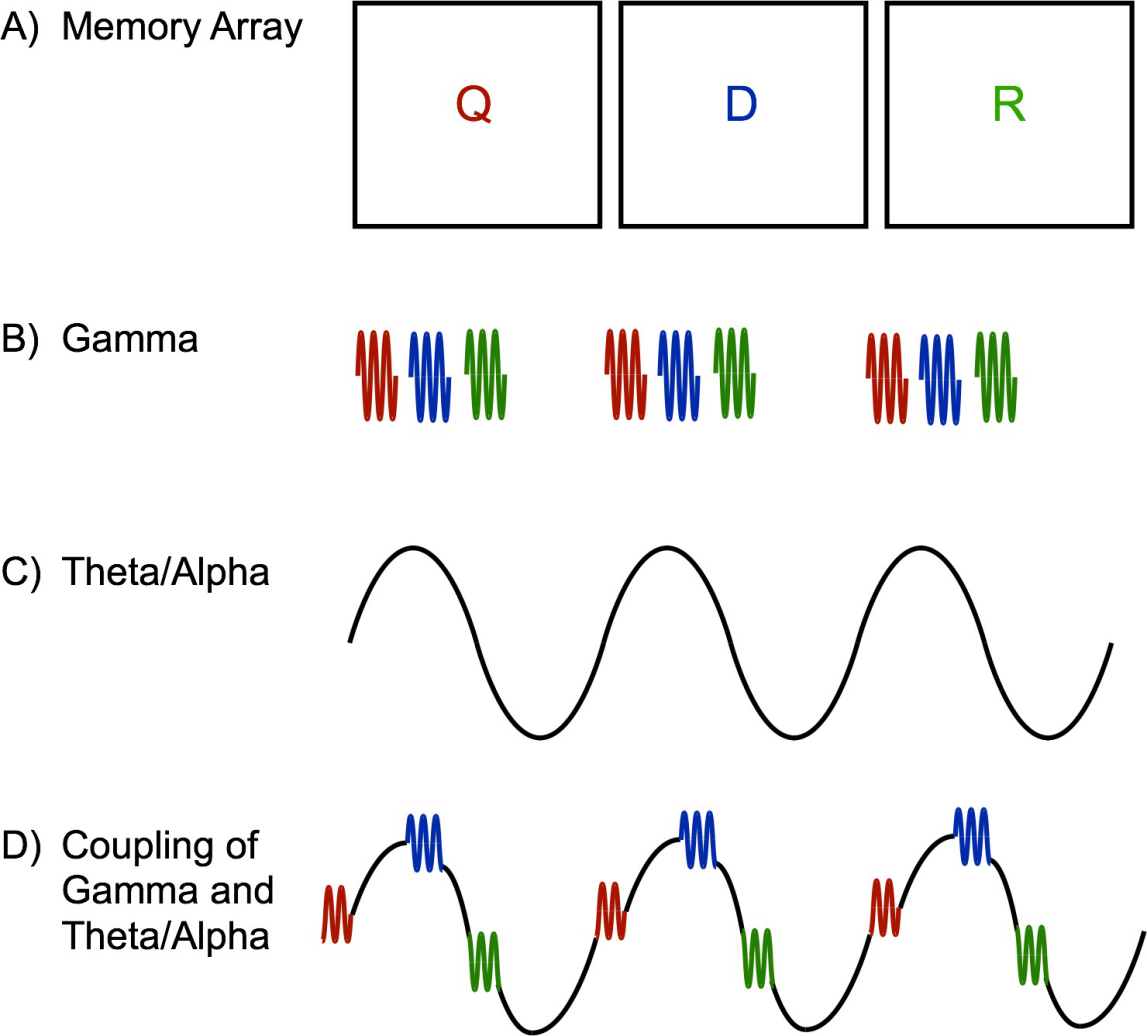
Prediction of the LIJ model. (A) Sternberg task with three letters presented sequentially in time. A brief delay and a memory probe follow the sequence of letters. At test, participants are presented with a letter and have to determine whether the new letter was part of the original memory array. In this figure, the letters are colored for illustrative purposes. In the actual task, all letters are presented in black. The phase amplitude coupled signal (D) is the sum of the gamma (B) and theta/alpha (C) oscillations. Each gamma burst represents the encoding of an individual letter (“Q” in red, “D” in blue, and “R” in green). The gamma burst that represents each individual letter is located in the same part of the theta/alpha phase. Additionally, the order of the gamma bursts that represents each individual letter follow the order in which the letters were presented in time, e.g., “Q”–“D”–“R.” LIJ, Lisman/Idiart/Jensen.

Oscillations at different frequencies often interact with each other in various ways (Fig 2). One common way is referred to as phase amplitude coupling in which the amplitude of a high-frequency oscillation is modulated as a function of the phase of a lower frequency oscillation. Previous work has shown that phase amplitude coupling happens throughout the brain, including in the hippocampus [22], basal ganglia [23], and neocortex [24]. The LIJ model of cortical phase coding [21] proposes that gamma and theta/alpha oscillations interact to maintain multiple object representations simultaneously by segregating them in different parts of the phase of the low-frequency wave. Specifically, individual objects held in working memory are represented by unique bursts in the gamma frequency band that occur in line with different moments of the theta/alpha band. For example, one object may be represented in a gamma burst that is coincident with the peak of the theta wave, while another object may coincide with the trough of the wave. Thus, multiple objects can be coded simultaneously without confusion because they are each represented in distinct portions of phase space. An exciting implication of this model is that it naturally proposes a potential explanation of why working memory is so limited to begin with. That is, it clearly articulates which neural resource actually limits working memory: phase space. There are only a certain number of distinct gamma bursts that can fit within a single cycle of theta/alpha, which sets a potential limit for how many representations can be kept separated at once. When more items are attempted to be stored, the representations overlap in phase space, which results in confusion errors. While the LIJ phase coding model is compelling and provides a clear explanation of capacity limits, obtaining empirical evidence for this model has been challenging. This is, in part, because gamma activity is difficult to measure from scalp-based electroencephalography (EEG) and magnetoencephalography (MEG) methods. The skull and scalp are essentially low-pass filters that greatly limit our observations of high-frequency activity, such as gamma.

## Evidence for phase code model of working memory

One of the first studies to provide evidence in favor of phase-dependent neuronal coding was conducted with population recordings in the prefrontal cortex of monkeys [25]. In this study, the monkeys were presented with a sequence of two images that they had to remember over a short delay. Following this delay, three images appeared on the screen; one was a novel image and the other two were the images from the beginning of the trial. The monkeys’ task was to look at the image that was presented first within the sequence. This task required that the monkeys remember both the images and the sequence in which they were presented. They found that the two remembered images were represented in distinct locations in phase space. Additionally, the image that was presented first in the sequence was located earlier in phase space. This study provided some of the first evidence that individual objects can be represented at distinct phases in an oscillation; however, it did not show that different frequency oscillations interact to maintain information in working memory.

A new, exciting study by Bahramisharif and colleagues (2018) in this issue of *PLOS Biology* extends these findings to recordings in the human brain and finds evidence in support of the LIJ model of phase coupling [3]. Individuals who have epilepsy that is not responding to traditional forms of treatment often undergo surgery in which electrocorticography (eCoG) grids are implanted on the surface of their brain. Medically, these eCoG grids are used to determine the locus of their seizure activity. However, when patients give consent, they can also volunteer to participate in scientific studies in which electrical activity on the surface of their brain can be recorded while they perform cognitive tasks. In this new study by Bahramisharif and colleagues (2018), they recorded electrical activity from 15 patients with eCoG grids implanted in their occipital and temporal lobes.

While recording activity from these eCoG grids, Bahramisharif and colleagues [3] presented the patients with serial sequences of three random letters that they had to remember over a blank delay. They were immediately presented with a single letter, and they had to determine whether that letter was part of the original memory sequence. They examined neural activity at “letter-selective” cortical sites, which are areas that show dissociable patterns of activity for individual letters. Previous research has illustrated that the amplitude of gamma activity, which is linked to theta oscillations, can distinguish the identity of a currently viewed letter [26]. In these “letter-selective” cortical sites, Bahramisharif and colleagues [3] found that each of the three letters were encoded as bursts of gamma activity at distinct positions in the phase space of the theta/alpha band. Additionally, the position of each item in the phase space depended on the position in the list when the item was presented. For example, if patients were presented with the sequence “Q, D, R,” the “Q” would be encoded in an earlier part of the phase space than the “D” and the “R.” Thus, the phase coding itself provided a cue regarding the temporal order of the memory stimuli. These results provide clear evidence that individual items can be represented at distinct phases in an oscillation in the human brain. Given the rarity of access to direct neural recordings in humans and the clarity of their findings, these results provide the strongest support for the LIJ model of cortical phase coding [21] to date.

## Remaining questions

The findings from Bahramisharif and colleagues [3] help us better understand how information is maintained in working memory. In particular, they provide a clear and compelling demonstration that information being actively held in working memory can be represented in distinct phase positions within an ongoing oscillation and thus satisfy a key prediction of the LIJ model. However, their experiment does not directly test how the limit in working memory capacity arises, as they had patients maintain information that was below working memory capacity. Yes, there does appear to be clear phase coding in this instance, but is that the reason why capacity is so limited? Another paper [27] investigated how the number of items in memory (one, two, or four items) influenced phase coupling in the human brain. They found that as the number of items held in mind increased, the gamma frequency was modulated by lower frequency theta band activity. Their results are consistent with the idea that longer cycles are required to maintain more items. However, without sequences that exceed putative capacity limits, it is difficult to determine if the limit in working memory capacity arises because of the limit in phase space. A clear prediction of the LIJ model in such a scenario is that, when the memory list exceeds capacity, there would be no remaining unused phase space. Nevertheless, the current work [3], in conjunction with Axmacher and colleagues (2010) [27], provides a clear blueprint for testing this ultimate prediction from the LIJ model.

One additional challenge for the LIJ model is to provide evidence that phase coding in working memory can generalize to instances in which multiple memory items are presented simultaneously. The current and prior demonstrations of phase coding have relied on paradigms in which the items are presented individually. This raises some questions about whether the observed phase coding is a natural coding schematic or a consequence of the serial presentation of the memoranda. Considering that the same working memory limits are observed even when items are presented simultaneously, evidence for phase-coded representations in these situations would go a long way toward demonstrating that the LIJ model could account for a wide swath of working memory–related phenomena.

Understanding how working memories are represented in the brain and why it is limited could greatly advance our understanding of intelligence and other higher level cognitive functions. While future work will be necessary to fully substantiate exactly how the limit in working memory arises, the new Bahramisharif and colleagues [3] study makes a clear step forward. It reveals how the brain segregates online memories to keep them from being confused and thus puts us one significant step closer to potentially understanding the fundamental capacity limit of the mind.

## Abbreviations

eCoG: electrocorticography
EEG: electroencephalography
LIJ: Lisman/Idiart/Jensen
MEG: magnetoencephalography
